# Impact of Ethical Leadership and Organizational Culture on Nursing Personnel's Organizational Commitment: A Cross-Sectional Study

**DOI:** 10.7759/cureus.86440

**Published:** 2025-06-20

**Authors:** Vasiliki Kourkouni, Petros Galanis, Konstantinos Athanasakis, Michael Igoumenidis

**Affiliations:** 1 Department of Nursing, University of Patras, Patras, GRC; 2 Clinical Epidemiology Laboratory, Faculty of Nursing, National and Kapodistrian University of Athens, Athens, GRC; 3 Department of Public Health Policy, School of Public Health, University of West Attica, Laboratory for Health Technology Assessment (LabHTA), Athens, GRC

**Keywords:** ethical leadership, nursing management, organizational commitment, organizational culture, turnover intention

## Abstract

Introduction

Nursing personnel's organizational commitment is a growing global concern, influencing retention in dynamic and high-demand healthcare environments. Ethical leadership and organizational culture both contribute to organizational commitment. Little is known about the interaction between these variables when they coexist, and whether the results vary between secondary and tertiary hospitals. This study aims to investigate the impact of ethical leadership and organizational culture on nursing personnel's organizational commitment in Greece and to examine whether this varies according to nursing institutions’ sizes.

Methods

A cross-sectional study was conducted, enrolling 724 nursing personnel from two secondary and two tertiary Greek hospitals. Data were collected from January to May of 2023. The tools used were the Ethical Leadership Scale, the Organizational Culture Profile, and the Three-Component Model of Commitment. Descriptive statistics, independent t-test, Spearman and Pearson correlation coefficient, and multivariate linear regression analyses were performed to explore the variables’ relationships.

Results

Ethical leadership was related to affective (β = 0.38, CI = 0.27-0.49), normative (β = 0.44, CI = 0.33-0.55), and continuance commitment (β = 0.21, CI = 0.09-0.31). The organizational culture factors of supportiveness (β = 0.31, CI = 0.15-0.47), decisiveness (β = 0.30, CI = 0.12-0.47), and team orientation (β = 0.30, CI = 0.13-0.46) were related to affective commitment. Innovation (β = 0.36, CI = 0.23-0.49), emphasis on rewards and growth (β = 0.21, CI =0.07-0.36), focus on results (β = 0.19, CI = 0.04-0.34), and team orientation (β = 0.25, CI = 0.07-0.42) were related to normative commitment. Secondary hospitals had better scores on commitment.

Conclusion

This study reinforces existing evidence on nursing personnel's organizational commitment being correlated to ethical leadership and organizational culture. Furthermore, it highlights that a smaller hospital size contributes to better outcomes. Nursing personnel's commitment is imperative and achievable through a strong organizational culture with benefits for nurses, patients, and organizations. These findings can guide health policies on hospital management style and nursing personnel leadership development education.

## Introduction

Commitment is a psychological and social attachment to someone or something in a social endeavor. Organizational Commitment (OCom) describes the exhibited level of members' commitment to the organization. It entails a belief in and acceptance of organizational values, a willingness to exert effort on behalf of the organization, and a strong desire to maintain membership in the organization [1]. Therefore, higher levels of OCom are related to increased performance and satisfaction, dedication to the organization’s goals, and lower absenteeism and turnover intention. According to the original framework of Meyer & Allen (1991), OCom has at least three separable components reflecting (a) a desire (affective commitment), (b) a need (continuance commitment), and (c) an obligation (normative commitment) to maintain employment in an organization [2].

OCom may be essential to the effectiveness and stability of health care organizations, and its relevance for nursing practice has been studied in various contexts, especially in relation to nursing personnel departing from the profession; nursing shortage worldwide necessitates a better understanding of factors that retain nurses to their organizations, and evidence shows that OCom is a major factor that can reduce nurses' turnover [3]. In addition, regardless of nurses’ turnover intentions, strong OCom has been positively correlated with better outcomes for patients and health care systems by enhancing productivity and performance and leading to better-quality care [4]. Nurses’ OCom is influenced by various factors related to personal characteristics, organizational context, characteristics of the job and work environment, as well as leadership and management [5]. Leadership is interrelated with organizational context and work environment; many studies indicate that appropriate leadership improves nurses' OCom and reinforces intent to stay [3, 6].

There are many different theories and categorizations of leadership styles, such as ethical leadership (EL), authentic leadership, or transformational leadership [7], and they all focus on ethical behavior as a prerequisite for effective leaders. Among these categories, the concept of EL has gained increased attention in the past decade. EL can be defined as the “demonstration of normatively appropriate conduct through personal actions and interpersonal relationships, and the promotion of such conduct to followers through two-way communication, reinforcement, and decision-making” [8]. Various studies have suggested that EL is related to work attitudes and behaviors, such as job satisfaction, well-being, performance, team spirit, and retention [9]. Within nursing contexts, evidence shows that EL could contribute to the mitigation of turnover intention and to commitment [10,11], as it leads to an organizational culture of excellence with reduced costs, enhanced patient experience, and clinician joy at work [12].

Organizational Culture (OCul) is a management concept that describes the basic assumptions about the world and the values that guide life in organizations [13]. Simply put, it is the personality of an organization that makes people feel good or bad about their working environment. Each organization’s culture is relatively unique, malleable, and subject to continual changes [14]. Furthermore, within an organization's culture, multiple subcultures may coexist, differentiated by factors such as the presence of various departments with distinct functional roles or the diversity of professional groups [13]. OCul significantly influences crucial aspects of the nursing profession, including retention [15], work-related stress [16], and the provision of quality care [17]. Studies suggest that OCul is strongly correlated to nursing leadership and organizational commitment [18], but available evidence is scarce.

In the context of the continuing shortage of nursing personnel worldwide and the need to identify appropriate health and labor policy reforms, the purpose of this study is to examine the impact of EL and OCul on nursing personnel’s OCom, enhancing the relevant literature. As a secondary aim, this study examines whether the impact of EL and OCul on OCom varies according to nursing institutions’ sizes. The expected results can guide managers to understand the crucial factors that influence nursing personnel’s OCom and subsequently design interventions that will enhance EL and OCul.

## Materials and methods

Design

A descriptive correlational, cross-sectional study was conducted between January and May 2023 in two secondary hospitals (≤300 beds) and two tertiary hospitals (>300 beds), all located in urban areas of Greece. The secondary hospitals are located in smaller urban areas compared to the tertiary ones. Of the tertiary hospitals, one is public and the other is university-affiliated.

Sampling and recruitment 

Sample Size and Power

Convenience sampling was used, which included all clinics of the participating hospitals. Participants were nurses and nursing assistants, working full-time in any clinical department. Inclusion criteria were their employment in the organization for at least 1 year (sufficient period to fully understand the OCul), their cooperation, and acceptance to participate after being informed. 800 questionnaires were distributed. 732 questionnaires were returned (response rate 91.5%). Incomplete questionnaires were removed. The final sample consisted of 724 (90.5%) valid returned questionnaires. Considering a low effect size (f2 = 0.05) of EL and OCul on OCom, the number of independent variables (16 in total), a confidence level of 95%, and a margin of error of 5%, the required sample size was estimated at 652 nursing personnel at least. The sample size of nursing personnel used in the research was 724 (>11.04% of the required sample size).

Data collection

Data was collected using self-administered questionnaires, which included socio-demographic and professional factors, as well as three pre-constructed self-report tools to assess EL, OCom, and OCul.

EL was assessed using the Ethical Leadership Scale (ELS), developed by Brown et al. (2005) [8]. It comprises 10 items rated on a five-point Likert scale. Cronbach's alpha was 0.90. Higher scores indicate greater EL behavior. The ELS items were designed to tap into the entire domain of ethical leadership and are applicable to leaders at all organizational levels. The scale was culturally adapted and validated in Greek by Kourkouni and Igoumenidis (2022) [19].

OCul was assessed using the Organizational Culture Profile (OCP) questionnaire, developed by O’Reilly et al. (1991) [20]. The OCP examines the extent to which person-culture fit is associated with individual commitment, satisfaction, and longevity within an organization [20]. It comprises 54 items classified into eight factors: focus on results, emphasis on rewards/growth, supportiveness, team orientation, innovation/risk taking, attention to detail, decisiveness, and aggressiveness/competitiveness. Responses are rated on a five-point Likert scale. Cronbach's alpha ranged from 0.84 to 0.90. It was culturally adapted and validated in Greek by Bellou (2007) [21].

Finally, Organizational Commitment was assessed using the revised version of the Organizational Commitment Questionnaire (OCQ), by Meyer et al. (1993) [22]. It comprises 18 items rated on a seven-point Likert scale. The responses reflect the three components of commitment: Normative, Affective, and Continuance Commitment, which describe employees' emotional and moral attachment to their organization/department. The OCQ examines the joint effects of this three-component framework on employees’ behavior, which can exert independent (and possibly interactive) effects on a particular behavior [2]. Cronbach's alpha ranged from 0.60 to 0.80. It was culturally adapted and validated in Greek by Tsolaki et al. (2013) [23].

Licenses for using these tools have been secured from their respective authors. Appropriate attribution and citation have been provided for each questionnaire.

Data analysis

Numbers and percentages were used to present categorical variables. Also, mean, median, standard deviation, minimum value, and maximum value were used to present continuous variables. The Kolmogorov-Smirnov test was used to evaluate the distribution of continuous variables. It was found that continuous variables (scores on scales) followed a normal distribution. EL and OCul were the independent variables, while OCom was the dependent variable. Socio-demographic variables were considered as potential confounders and were eliminated by multivariate analysis. First, bivariate analysis was conducted between independent variables and scores on three OCom's factors, which included independent samples t-test, Spearman’s correlation coefficient, and Pearson’s correlation coefficient. Subsequently, a multivariate linear regression analysis was performed to remove confounders from socio-demographic variables. Variables that were statistically significant (p < 0.05) in the multivariable models are presented. In that case, adjusted coefficients beta, 95% confidence intervals, and p-values are presented. Two-tailed p-values <0.05 were considered statistically significant. For the statistical analysis, IBM SPSS 21.0 was used (IBM Corp., Armonk, USA).

Ethical considerations

Permissions to conduct the study were secured from the hospitals’ ethics committees. Nursing personnel were informed about the study’s purpose. Their participation was voluntary and anonymous. A consent form was attached. Collected questionnaires were placed in an opaque folder at a place where only the researchers had access. The process ensured, in the best possible way, the participants' informed consent, anonymity, and the information's confidentiality.

## Results

Demographic and job characteristics are presented in Table 1. Most participants were female (n = 605; 83.6%) and aged ≥45 years (n = 376; 51.9%). The majority held nursing degrees (n = 514; 71%). A total of 101 participants (13.9%) held a master's or doctorate degree, while 210 (29%) were nursing assistants. Most participants had less than 21 years of work experience at the current hospital (n = 378; 52.2%), and half of them worked in hospitals with ≤300 beds (n = 363; 50.1%).

**Table 1:** Socio-demographic characteristics of nursing personnel (N=724)

Characteristics	Ν	%
Gender		
Male	119	16.4
Female	605	83.6
Age in years		
<45	348	48
≥45	376	51.9
Education level		
Νursing assistants	210	29
Bachelor in nursing	413	57.1
MSc	93	12.8
Ph.D	8	1.1
Years of work experience at the current hospital		
1-10	190	26.2
11-20	188	26
21-30	218	30.1
≥31	128	17.7
Type of hospital		
Secondary (≤300 beds)	363	50.1
Tertiary (>300 beds)	361	49.9

Descriptive statistics for the study scales are presented in Table 2. The mean value for the ELS was 3.8 (SD = 0.8), with a median value of 3.9, indicating a moderate to high level of EL behavior. Mean scores for the eight factors of the OCP ranged from 2.8 to 3.3. Higher scores were observed for the factors supportiveness, emphasis on rewards/growth, focus on results, team orientation, attention to detail, and decisiveness. Aggressiveness/competitiveness and innovation/risk taking recorded lower scores. Mean scores for the Continuance, Affective, and Normative Commitment subscales were 4.7, 4.2, and 4.0, respectively, indicating moderate OCom levels. Detailed results from bivariate analysis between independent variables and OCom are presented in Table 3.

**Table 2:** Descriptive statistics for the study scales

Scales	Mean	Standard Deviation	Median	Min	Max
Ethical Leadership	3.8	0.8	3.9	1	5
Organizational culture					
Innovation/risk taking	2.8	0.9	2.8	1	5
Supportiveness	3.3	0.9	3.3	1	5
Decisiveness	3.1	0.8	3.3	1	5
Emphasis on rewards/growth	3.3	1	3.3	1	5
Focus on results	3.3	0.9	3.3	1	5
Attention to detail	3.2	0.9	3.3	1	5
Team orientation	3.3	0.9	3.3	1	5
Aggressiveness/competitiveness	3.0	0.8	3	1	5
Organizational Commitment					
Affective Commitment	4.2	1.4	4.2	1	7
Continuance Commitment	4.7	1.2	4.7	1	7
Normative Commitment	4.0	1.5	4	1	7

**Table 3:** Bivariate analysis between independent variables and OCom AC: Affective Commitment; CC: Continuance Commitment; NC: Normative Commitment; Ocom: Organizational Commitement ^a ^independent samples t-test; ^b ^Spearman’s correlation coefficient; ^c ^Pearson’s correlation coefficient

Independent variables	Grading AC			Grading CC			Grading NC		
	Mean score	SD	p-value	Mean score	SD	p-value	Mean score	SD	p-value
Gender			0.9^a^			0.2^a^			0.9^a^
Female	4.2	1.4		4.7	1.2		4.0	1.5	
Male	4.1	1.6		4.5	1.3		4.0	1.6	
Education level		-0.2^b^	<0.001^b^		-0.1^b^	0.1^b^		-0.1^b^	<0.001^b^
Years of work experience		0.1^b^	<0.001^b^		0.1^b^	0.1^b^		0.2^b^	<0.001^b^
Type of hospital			<0.001^a^			<0.001^a^			<0.001^a^
Secondary ≤300 beds	4.4	1.4		4.8	1.2		4.3	1.5	
Tertiary >300 beds	3.8	1.4		4.5	1.3		3.7	1.4	
Ethical Leadership		0.5^c^	<0.001^c^		0.2^c^	<0.001^c^		0.5^c^	<0.001^c^
Innovation/risk taking		0.5^c^	<0.001^c^		0.1^c^	0.04^c^		0.6^c^	<0.001^c^
Supportiveness		0.6^c^	<0.001^c^		0.1^c^	0.07^c^		0.6^c^	<0.001^c^
Decisiveness		0.6^c^	<0.001^c^		0.1^c^	0.1^c^		0.6^c^	<0.001^c^
Emphasis on rewards/growth		0.6^c^	<0.001^c^		0.1^c^	0.004^c^		0.6^c^	<0.001^c^
Focus on results		0.6^c^	<0.001^c^		0.1^c^	0.1^c^		0.6^c^	<0.001^c^
Attention to detail		0.5^c^	<0.001^c^		0.1^c^	0.1^c^		0.5^c^	<0.001^c^
Team orientation		0.6^c^	<0.001^c^		0.1^c^	0.1^c^		0.6^c^	<0.001^c^
Aggressiveness/competitiveness		0.1^c^	0.2^c^		0.02^c^	0.6^c^		0.1^c^	0.1^c^

Afterwards, multivariable linear regression analysis was performed to eliminate confounders (Table 4). Affective Commitment was related to higher EL (β = 0.38, CI = 0.27-0.49), supportiveness (β = 0.31, CI = 0.15-0.47), decisiveness (β = 0.30, CI = 0.12-0.47), and team orientation (β = 0.30, CI = 0.13-0.46). Normative Commitment was related with increased EL (β = 0.44, CI = 0.33-0.55), innovation/risk taking (β = 0.36, CI = 0.23-0.49), emphasis on rewards/growth (β = 0.21, CI =0.07-0.36), focus on results (β = 0.19, CI = 0.04-0.34) and team orientation (β = 0.25, CI = 0.07-0.42). EL predicted Continuance Commitment (β = 0.21, CI = 0.09-0.31). Employees in hospitals with ≤ 300 beds recorded greater OCom across all three components.

**Table 4:** Multivariable linear regression with organizational commitment as the dependent variable CI: Confidence interval

Dependent variable	Adjusted coefficient beta	95.0% CI for beta	p-value
Independent variables			
Grading Affective Commitment			
Ethical leadership	0.38	0.27-0.49	<0.001
Supportiveness	0.31	0.15-0.47	<0.001
Decisiveness	0.30	0.12-0.47	0.001
Team orientation	0.30	0.13-0.46	<0.001
Hospital ≤300 beds	0.21	0.06-0.36	0.006
Grading Continuance Commitment			
Ethical leadership	0.21	0.09-0.31	<0.001
Hospital ≤300 beds	0.27	0.08-0.45	0.004
Grading Normative Commitment			
Ethical Leadership	0.44	0.33-0.55	<0.001
Innovation/risk taking	0.36	0.23-0.49	<0.001
Emphasis on rewards/growth	0.21	0.07-0.36	0.003
Focus on results	0.19	0.04-0.34	0.014
Team orientation	0.25	0.07-0.42	0.005
Hospital ≤300 beds	0.31	0.15-0.46	<0.001

## Discussion

Interest in OCom is growing in the nursing literature. Previous studies have individually examined the influence of EL [24, 25] and OCul [18] as optimal predictors of OCom. However, less attention has been paid to the combined effect of these factors on OCom, which constitutes the rationale for the present study.

According to the findings, EL and OCul significantly affect OCom. EL was found to increase all three components of OCom. These results are in accordance with previous studies [24, 26]. In the cases of Affective and Normative Commitment, EL is one of the factors that accounts for 51.4% and 52% of the variance, respectively. In contrast, regarding Continuance Commitment, EL is one of only two variables explaining just 3.4% of the variance, and is not influenced by OCul factors. Continuance Commitment reflects a need. Employees with high levels of Continuance Commitment recognize the perceived costs of leaving and remain in their jobs primarily because they feel they have no alternatives. Therefore, employees in settings with guaranteed employment, such as the public sector, may exhibit lower performance levels [2], and thus, reduced commitment to the organization's goals. Based on the results, the variable Continuance Commitment does not appear to be significantly influenced by either OCul or EL factors.

In OCul, enhancing the factors support, team orientation, and decisiveness increases nurses’ Affective Commitment. Indeed, Affective Commitment is influenced by work experience and reflects values ​​such as fairness and expectations; members are oriented towards organizational effectiveness and well-being, they share common values, and develop mutually beneficial relationships [2]. The cultural factors emphasizing rewards/growth, innovation/risk taking, focus on outcome, and team orientation enhance Normative Commitment. These elements nurture employees through reciprocity, promote collectivism over individualism, and encourage them to remain in the organization even when other attractive alternatives are available [2].

Competitiveness/aggressiveness and attention to detail were not related to OCom. There is no corresponding correlation study. However, evidence shows that nursing culture, which is an occupational subculture of OCul, is strongly correlated to Affective Commitment [18]. Furthermore, significant correlations have been found between nurses’ perception of the overall ethical work climate, their perceived organizational support, and commitment [27]. However, in the present study, EL focused on clinics, rather than the overall ethical work climate, because nurses are more directly influenced by head nurses, an influence that varies between clinics, and indirectly by senior leadership. Also, this study examined eight OCul factors, not just supportiveness. Similar associations between EL, OCul, and commitment have been observed in previous studies among healthcare professionals [28-30].

Regarding the organization size, employees in secondary hospitals reported greater OCom across all three components, compared to those in tertiary hospitals. No corresponding study has been found to correlate Ocom with the hospital size. However, a study in Greek hospitals confirms cultural differences between secondary and tertiary hospitals; secondary hospitals scored higher on OCul factors that reflect a person-culture fit [17]. These factors are associated with better commitment [20]. Moreover, a study found lower perceived EL and commitment among nurses in university hospitals in large cities compared to those in smaller local hospitals [26], highlighting the challenges posed by the complex organizational structure of university-affiliated hospitals and the rapid, impersonal pace of urbanization.

The negative results for tertiary hospitals may be attributed to the increasing workload and the system's strenuous demands they face [17]. Leadership behaviors are often influenced by variables such as the size of the organization. It is more likely that in large organizations, administrative responsibilities tend to increase, jobs become more demanding, and opportunities for interaction and effective interpersonal relationships among staff may be limited. Participation in group meetings, as well as individualized teaching, guidance, and support, may also be restricted. Therefore, it is possible that the size of the organization affects OCom.

Nursing personnel interact extensively with patients. Their behaviors and attitudes directly impact complex therapeutic care relationships. Enhancing nursing personnel's commitment requires redefining healthcare services, as well as fostering comprehension and motivation among human resources personnel. A strong ethical OCul can unify an organization. It is transmitted from leadership to members, limits deviant behavior, and increases nursing personnel's satisfaction and commitment [11].

Strategies to support and enhance EL and OCul in nurse leaders are suggested. Educational interventions that address ethical issues in organizational processes can improve interpersonal relationships and help manage workplace challenges. Ideally, empowerment courses should be integrated into curricula [12]. Reward systems represent a key component of effective interventions [8]. Senior organizational and governmental leaders must demonstrate commitment to implementing these interventions.

Limitations and recommendations

Convenience sampling is an obstacle to drawing general conclusions. The majority of the sample is female. Being a cross-sectional study, findings are limited to the time period of the study. Also, nursing personnel's perceptions may differ in other public hospitals, so they cannot be generalized. However, the limitation of generalization does not limit the fact of the correlation between variables and OCom. Future research could examine the correlation of EL with the eight factors of OCul.

## Conclusions

Health care services provide the precious commodity of health operating within an extremely demanding environment shaped by economic, social, political, and medical technological developments and requirements. These factors often increase nursing personnel's intention to leave the profession. At the same time, in this rapidly evolving healthcare landscape, health professionals must protect the rights and dignity of patients by delivering high-quality, patient-centered care. Consequently, OCom plays a crucial role in human resource management. It determines nursing personnel's retention, work performance, productivity, and effectiveness, while also supporting the sustainability of health services and the achievement of the WHO's Sustainable Development Goals.

The current study highlights that EL and OCul significantly influence nursing personnel's OCom. Smaller hospitals appear to achieve better outcomes. Ethical leadership behaviors promote cultural values that enhance organizational function and foster an ethical OCul. These factors, in an interrelated manner, ensure correspondence and homogeneity between individual and organizational values and contribute to the organization's commitment to goals. There is an urgent need for a rigorous evidence-based approach to the education and training of head nurses in EL, as well as the adoption of an OCul that strengthens nursing personnel’s OCom and ultimately improves their retention. Leaders, as the cornerstone for the creation and maintenance of a quality health system, carry a social responsibility to address challenges and establish supportive organizational conditions for nursing personnel's sustained commitment.
